# Personalized treatment of malignant tumors during pregnancy

**DOI:** 10.1097/MD.0000000000029803

**Published:** 2022-06-30

**Authors:** E. Ulrikh, E. Kalinina, E. Dikareva, E. Komlichenko, O. Li, O. Zhamborova, I. Rizhinashvili, A. Dzharbaeva, I. Govorov, V. Artemenko, V. Bezrukikh, G. Salogub, T. Pervunina, A. Urmancheeva

**Affiliations:** a Personalised medicine center, Almazov National Medical Research Centre, St. Petersburg, Russia; b North-Western State Medical University named after I.I. Mechnikov, St. Petersburg, Russia; c National Medical Research Center of Oncology named after N.N. Petrov, St. Petersburg, Russia.

**Keywords:** cancer, hematologic malignancies, malignant tumors, pregnancy, solid tumors

## Abstract

The combination of pregnancy and cancer is a challenge for the patient and a problematic clinical dilemma for the doctor. In this retrospective observational cohort study, we have tried to analyze our experience in the management of such patients.

This review includes 41 patients with malignant neoplasms detected during pregnancy who received treatment at the Almazov National Medical Research Centre from 2015-2021.

The majority of patients received treatment during pregnancy (n=26, 63.4%): chemotherapy – 19 (46.3%) (in 2 cases in combination with surgery), surgical treatment – 7 (17, 1%) patients. In most cases, delivery was at term (n=28, 68.3%). All children born at term were mature and had no growth restriction, regardless of whether the mothers received treatment during pregnancy or not.

When detecting cancer during pregnancy, an immediate follow-up examination is required to assess the extent of the tumor and current fetal state. If pregnancy prolongation is requested, the treatment should not be postponed, except for systemic chemotherapy in the first trimester of pregnancy, pelvic radiation at any term.

## 1. Introduction

Over the past few years, there has been an increase in cancer incidence among pregnant women worldwide.^[1]^ The trend is partly due to the desire of modern women to postpone childbirth while prioritizing social well-being and career. The late age of the desired pregnancy may coincide with the onset of the malignant disease. Cancer associated with pregnancy is defined as a malignant neoplasm during pregnancy or the first year postpartum. It is considered that malignancy is found in 0.05-0.1% of all pregnancies, with the most common, are breast cancer, ovarian cancer, cervical cancer, Hodgkin and nonHodgkin lymphomas (NHLs).^[2–4]^ Although the combination of pregnancy and cancer is not rare, each case is challenging for the patient and clinician.^[3–5]^ When cancer is detected in a pregnant woman, the doctor usually faces a dilemma of setting the equilibrium between the benefits of providing proper management for the sake of the mother, on the 1 hand, and the possible harmful effects for the fetus, on the other. The issue becomes even more dramatic in the case of an aggressive disease when a treatment delay can lead to the death of both mother and fetus. The management strategy should be tailored for each patient according to the entire clinical picture, stage of the disease, gestational age and, last but not least, the patient’s desire to preserve the pregnancy. Ideally, the management of such patients should be carried out by a multidisciplinary team, including oncologists, obstetricians, neonatologists, endocrinologists, hematologists, surgeons and other specialists. In addition, it seems necessary to form a long-term commitment to the treatment of the patient.^[6–8]^ Despite a growing body of clinical data, there is a lack of standard algorithms for diagnosis and treatment, which necessitates collecting the observations from the clinics worldwide and further analysis on the supranational level.

Our main objective was to investigate the prevalence of cancer among pregnant women and to analyze existing treatment options.

## 2. Materials and Methods

It was a retrospective observational cohort study.

During 2015–2020, 193 pregnant women with either a history of malignancy or a newly diagnosed cancer were delivered at the perinatal centre of the Almazov National Medical Research Centre. The average age of women was 33.3 ± 5.5, ranging from 21 to 48 years. Ninety-five (49%) patients were diagnosed with haematological malignancy, while others (51%) had solid tumors. By the time of pregnancy, 141 (73%) women had previously been treated for cancer and had clinical remission. In 41 (21%) cases, cancer was detected during pregnancy. In 11 patients (6%), pregnancy coincided with myeloproliferative disease. In the current article, we would like to focus on patients diagnosed with malignancy during pregnancy.

Relevant medical records were retrieved and analyzed according to the following parameters: age, weeks of gestation, background clinical information, type of malignancy, management options (including adjuvant and neoadjuvant therapy), pregnancy outcomes, fetal and maternal peripartum morbidity. All clinical data was analyzed according to clinical guidelines established in the Almazov National Medical Research Centre. In order to protect patients’ integrity and privacy additional measures were taken, such as processing deidentified data. All patients signed informed consent on analyzing and publishing the clinical data. A separate review from the Institutional Board was not required.

Since the primary objective was to summarize and report our experience in managing pregnant women with malignancies, we mainly utilized descriptive statistics.

## 3. Results

Forty-one pregnant women who were diagnosed with cancer during pregnancy were selected for the final analysis. The average age of the patients was 31.9 ± 6 years, ranging from 22 to 45 years. The pregnancy occurred following spontaneous conception in 39 cases (95.1%), the ovulation induction in 1 case (2.4%), as a result of in vitro fertilization in another pregnancy (2.4%). All patients requested pregnancy prolongation and underwent diagnostic measures to assess the tumor spread and fetal status. Of them, 25 patients were diagnosed with solid tumors and 16 patients with oncohaematological diseases (Table 1).

**Table 1:** The type of malignancy diagnosed during pregnancy.

Type of malignancy	(n = 41)	(n%)
Hemoblastosis	16	39.0
Hodgkin lymphoma	8	19.5
Non-Hodgkin lymphoma	7	17.1
Acute lymphoblastic leukemia	1	2.4
Solid tumors by site	25	61.0
Thyroid	5	12.2
Breast	5	12.2
Ovary	4	9.8
Cervix	3	7.3
Kidney	2	4.9
Brain	1	2.4
Melanoma	1	2.4
Colon	1	2.4
Stomach (gastric)	1	2.4
Soft tissue sarcoma	1	2.4
Retroperitoneal/intraabdominal sarcoma	1	2.4

### 3.1. Hemoblastosis

The diagnosis was established based on morphological examination of tumor tissue and/or bone marrow using immunohistochemical, cytogenetic and molecular genetic methods following the WHO classification.^[9]^ In order to further assess tumor spread, the patients underwent magnetic resonance imaging (MRI) and/or ultrasound; the staging was carried out per the Ann-Arbor criteria, modified by the Cotswolds. If necessary, computed tomography (CT) scans of the chest and soft neck tissues of the neck with fetal protection was performed. Whole-body positron emission tomography (PET) with 18F-fluorodeoxyglucose was performed in the postpartum period to assess the distant metastases and/or the effectiveness of treatment.

Hodgkin lymphoma (HL) was diagnosed in 8 patients, with 5 of them receiving chemotherapy during pregnancy (Table 2).

**Table 2:** Pregnant with lymphomas, leukemia (n = 16) hematologic diseases/lymphoproliferative disorders.

No	Diagnosis	Age	Time of diagnosis (weeks of gestation)	Treatment during pregnancy	Labor (weeks)	Mode of delivery	Newborn (Apgar)
1	HL II В	29	16/17	ABVD N2	39/40	Spontaneous vaginal	7/8
2	HL II A	31	29/30	ABVD N2	37/38	Spontaneous vaginal	8/9
3	HL II B	30	21/22	ABVD N4	38/39	Spontaneous vaginal	8/9
4	HL II A	24	24/25	ABVD N4	37/38	Labor induction, vaginal	5/7*
5	HL II B	30	23/24	ABVD N3	39/40	Spontaneous vaginal	8/9
6	HL III AХ	31	37/38	Not performed	38/39	Spontaneous vaginal	8/9
7	HL II A Recurrence	23	6/7	Refusal of treatment	37/38	Cesarean section	8/9
8	HL IV B Recurrence	29	37/38	Not performed	38/39	Spontaneous vaginal	8/9
9	NHL DLBCL IVВ	34	25/26	*R*-CHOP N3	35/36	Spontaneous vaginal	8/9
10	NHL DLBCL II A	27	32/33	*R*-CHOP N2	37/38	Spontaneous vaginal	8/9
11	NHL DLBCL II AX	22	21/22	*R*-CHOP N3	29/30	Spontaneous vaginal	Antenatal fetal death
12	NHL DLBCL II AX	28	37/38	Not performed	39/40	Spontaneous vaginal	7/8
13	NHL DLBCL IV B	31	20/21	*R*-DA- EPOCH N1	24/25	Spontaneous vaginal (premature rupture of the membranes)	5/7
14	NHL T-Cell Lymphoma I A	23	12/13	Surgical treatment at GW 12/13 (intestinal resection). Refusal of the therapy	39/40	Spontaneous vaginal	7/8
15	NHL T-Cell Lymphoma IIA	34	22/23	ALL-ВFM 2009, block IB	28/29	Cesarean section, uterine bleeding, hysterectomy	6/8
16	Acute Lymphoblastic Leukemia IIIВ	25	26/27	ALL-ВFM 2000	36/37	Labor induction, vaginal	6/8*

ABVD = doxorubicin+bleomycin+vinblastine+dacarbazine, ALL = Acute Lymphoblastic Leukemia, ALL-ВFM = cytarabine, cyclophosphamide and intrathecal administration of methotrexate, DLBCL = Diffuse Large B-Cell Lymphoma, HL = Hodgkin’s Lymphoma, NHL = Non-Hodgkin’s Lymphoma, *R*-CHOP = rituximab+cyclophosphamide+doxorubicin+vincristine+prednisolone, *R*-DA-EPOCH = etoposide+prednisone+vincristine+cyclophosphamide+doxorubicin+rituximab.

*– Due to a complicated obstetric situation (the onset of fetal hypoxia, use of the vacuum extractor).

In patient No. 1, the therapy for newly diagnosed HL was urgently initiated at the beginning of the second trimester due to increased tumor volume, signs of mediastinal compression and clinical features of respiratory failure. The optimal response (partial response with a 75% reduction in tumor mass according to MRI) was achieved after 2 courses of ABVD (doxorubicin, bleomycin, vinblastine, and dacarbazine).^[10]^ A healthy newborn (Apgar score 7/8) was delivered spontaneously at 39 gestational weeks (GW). On the 20th day of the postpartum period, a complete metabolic response was verified by PET/CT.

Patients (No. 2-5) with HL diagnosed in the second or early third trimester of pregnancy underwent 2 to 4 courses of therapy (ABVD) without any serious adverse events. All of them delivered healthy full-term newborns.

Three out of 8 patients with HL (No. 6–8) did not receive therapy. In 1 case (No. 6), the diagnosis of HL III AH was established at GW 37, which, in the absence of urgent indications for therapy, made it possible to postpone the start of treatment until the postpartum period.

Two patients (No. 7–8) had a relapse of HL during pregnancy. In patient No. 7, pregnancy occurred following 4 lines of polychemotherapy, autologous and allogeneic stem cell transplantation, and immunotherapy with the PD1 inhibitor nivolumab. The patient refused to terminate the pregnancy, which was recommended in the first trimester and was admitted at GW 36/37. Considering the extent of tumor spread (lower extremity paraparesis, massive tumor spread within the chest, spinal canal and compression of the spinal cord at the Th7-Th11 level), delivery was performed by cesarean section (CS) at 37 weeks. A live full-term boy was born with an Apgar score of 8/9 points.

Patient No. 8 was diagnosed with disease progression (axillary lymph nodes enlargement, fever, weight loss 5 kg, heavy night sweats) following 4 chemotherapy courses, sought medical help at 37 weeks of gestation and was delivered at 38 weeks.

Out of 7 patients with NHLs, 5 were diagnosed with diffuse large B-cell lymphoma (DLBCL), 1 had T-cell lymphoma associated with enteropathy, and another had T-lymphoblastic lymphoma/leukaemia (Table 2).

Despite the appearance of B symptoms (fever, drenching night sweats, loss of more than 10 % of body weight over 6 months), clinical features of respiratory failure before the onset or during the early stages of pregnancy in 4 out of 5 patients with DLBCL, the diagnosis was established only in the II or III trimesters of pregnancy. Until the final histological verification of the diagnosis, corticosteroid therapy (dexamethasone 10-20 mg/m^2^) was carried out.

Three patients received *R*-CHOP (rituximab, cyclophosphamide, doxorubicin, vincristine, and prednisolone) therapy.^[11]^ Patient No. 9 was admitted at GW 25, despite the onset of the symptoms 4 months before pregnancy. The severity of the condition was primarily due to respiratory failure, which occurred because of the bronchial compression by the mediastinal “tumor” measuring 21.5x15.6x15.1 cm, with infiltration of the mediastinal and costal pleura, lungs, invasion of the anterior chest wall with spread into the soft tissues of the neck, compression of the superior vena cava. Due to a life-threatening condition, *R*-CHOP chemotherapy was started with a 40% reduction in tumor masses after the first cycle of chemotherapy. On the 12th day of *R*-CHOP No 3, preterm spontaneous labor occurred at GW 35, with a live premature girl with an Apgar score of 8/9 points.

Patient No. 10 was diagnosed at 32 weeks of gestation, 2 courses of therapy were carried out with a partial response (reduction of tumor masses of more than 70%), which made it possible to prolong pregnancy up to 37/38 weeks. The patient vaginally delivered a living full-term boy with an Apgar score of 8/9.

Patient No. 11 was diagnosed in the second trimester of pregnancy; at the onset of the disease, acute heart failure developed with the signs of cardiac tamponade. In total, three courses of therapy were carried out. On the fifth day after the end of the third course of therapy, antenatal fetal death occurred. Labor induction was performed at 29 weeks of gestation.

Patient No. 13 was diagnosed at 20/21 weeks of gestation. The *R*-DA-EPOCH (etoposide, prednisolone, vincristine, cyclophosphamide, doxorubicin and rituximab) was selected as the therapy mode.^[11]^ On the 12th day after the end of the therapy, due to premature rupture of the membranes, given the high risks of developing septic complications and ongoing cancer treatment, vaginal delivery was carried out at a gestational age of 24 weeks. A live premature boy was born, weighing 700 g, 29 cm long, with an Apgar score of 5/7 points. At the moment, the child grows and develops according to his age.

Patient No. 14 with NHL at 13 weeks of gestation underwent diagnostic laparotomy due to suspicion of an “acute abdomen” followed by intestinal resection, which confirmed diagnosis based on the results of the histological examination. The patient refused the proposed therapy and was delivered vaginally at the 40th week of a living full-term girl with an Apgar score of 7/8 points.

Patient No. 15 was diagnosed with NHL T-cell II-A at GW 22. Considering the high risk of progression of T-lymphoblastic lymphoma, the patient was advised early delivery and consequently high-dose specific polychemotherapy (PCT). However, taking into account the patient’s refusal, we chose to conduct a restraining course of PCT according to the ALL IC-BFM 2009 block IB (cytarabine, cyclophosphamide, and intrathecal administration of methotrexate) with exclusion from the 6-MR protocol until the desired time for delivery (which is determined as 28 weeks and the fetus weight of 850 grams). At GW 28/29, a live premature girl was born via CS, weighing 1060 g, 39 cm long, with an Apgar score of 6/8 points.

Patient No. 16, with a verified diagnosis of acute lymphoblastic leukaemia, variant BIII in the period 26/27 weeks of gestation, was treated according to the ALL-BFM 2000 protocol^[12]^ with the achievement of the Ist bone marrow remission. The developed complications (grade 3-4 postcytostatic pancytopenia and toxic hepatitis) required the termination of the 2nd induction phase of chemotherapy. However, preeclampsia occurred at GW 37 and a live premature boy with an Apgar score of 6/8 points was delivered vaginally.

The primary data on the management of pregnancy, delivery, the condition of newborns in haematological cancer patients are presented in Table 2.

### 3.2. Solid tumors

Solid tumors were found in 25 patients (61%): thyroid cancer (n = 5, 12.2%), breast cancer (n = 5, 12.2%), malignant ovarian tumors (n = 4, 9.8%), cervical cancer (n = 3, 7.3%), kidney cancer (n = 2, 4.9%). Tumors of the central nervous system (CNS), intestines, stomach, soft tissue sarcoma, retroperitoneal leiomyosarcoma and melanoma accounted for 1 case each (2.4%) (Table 1). In 7 patients, solid neoplasms were detected in the first trimester, thirteen in the second trimester, and 5 in the third trimester.

Breast cancer was detected in 5 pregnant patients. In 4 of them, it was diagnosed in the Stage III of the disease, and in 1 pregnant woman in the Stage II. In a patient with Stage II of the disease (Patient No. 17), the tumor was diagnosed at 33/34 weeks of pregnancy. Treatment was postponed for the postpartum period. The patient was delivered vaginally at GW 37. Due to the intrapartum complications (fetal hypoxia followed by vacuum extraction), the newborn had an Apgar score of 5/7 points. In Patient No. 18 with Stage III breast cancer, the tumor was detected at GW 25/26. The patient refused to undergo chemotherapy, and therefore the delivery was carried out at 34 weeks via CS with the primary aim to initiate therapy earlier. A live premature baby boy weighing 2350g, 43 cm tall with an Apgar score of 7/8 points was born. The remaining patients with Stage III of the disease underwent chemotherapy (2–4 courses) according to the scheme AC (doxorubicin, cyclophosphane) or EC (epirubicin, cyclophosphane) during pregnancy. These patients vaginally delivered full-term children with an Apgar score of 7/8-8/9 points.

Three patients were presented with ovarian cancer (2 of them had high-grade lesions, 1 had low-grade), 1 had nonepithelial tumor. In 2 patients, the tumor was detected in the first trimester of pregnancy and 2 in the second trimester. The laparoscopy was performed only in 1 patient with Stage I disease; in other cases, conversion to laparotomy with adnexectomy, resection of the omentum, and biopsy of the contralateral ovary. Subsequently, 2 patients with high-grade tumors received chemotherapy during pregnancy (TCb – paclitaxel, carboplatin). Patient No. 25, with a stromal cell tumor, refused to terminate pregnancy and treatment during pregnancy. She delivered vaginally (GW 38) a full-term baby with an Apgar score of 8/9 points. On the 8th day of the postpartum period, the tumor continued growth was diagnosed (ultrasound, CT, MRI), which necessitated abdominal hysterectomy and bilateral salpingo-oophorectomy. The histological evaluation confirmed the Sertoli-Leydig tumor in the contralateral ovary and parietal peritoneum.

In 1 patient with a high-grade ovarian tumor (Patient No. 22), fetal status deterioration occurred (intrauterine growth restriction (IUGR), oligohydramnios, decrease in uteroplacental blood). Therefore, it was decided to cancel the third course of chemotherapy and deliver the patient at GW 34. Following the CS, the uterus, adnexae and omentum were removed together with lymph nodes. A live premature newborn had an Apgar score of 7/7 points. Histologically, the contralateral ovary had high-grade carcinoma, with no detectable lesions within the omentum and lymph nodes. Treatment was continued after childbirth.

Patient No. 23, with high-grade ovarian carcinoma, was operated on at GW 22 and received 4 cycles of adjuvant chemotherapy according to the TCb scheme). A CS was done at GW 38 together with a hysterectomy and bilateral salpingo-oophorectomy, omentectomy. A live full-term boy was born weighing 3070g, 51 cm long, with an Apgar score of 8/9 points. Treatment was continued after childbirth.

Patient No. 24 had IA low-grade ovarian carcinoma, diagnosed following laparoscopy and removing the left adnexa, omentum, biopsy of the contralateral ovary at GW 22. At GW 36, a CS was performed due to placental abruption, with no signs of progression. A healthy girl was born weighing 2910 g, 50 cm long, with an Apgar score of 7/8 points.

Three patients were diagnosed with cervical cancer during pregnancy. In patient No. 26, a neoplasm (T2bN0M0) was detected in 25/26 weeks of pregnancy, 2 cycles of chemotherapy were performed according to the TP scheme (paclitaxel, cisplatin) with a positive effect (tumor regression by 40%). At 37/38 weeks, a CS and radical hysterectomy (Piver III) was performed. A healthy full-term boy was born with an Apgar score of 8/9 points.

In patient No. 27, a large cervical lesion (6 cm) was detected at GW 18. However, she sought medical care only ten weeks later due to a progressively deteriorating condition. Considering the presence of IUGR, 3^rd^ stage violations of the uteroplacental blood flow and the onset of fetal hypoxia, a delivery was performed by CS with temporary bilateral embolization of the uterine arteries at GW 29. A live premature baby boy weighing 940g and 35 cm long was born with an Apgar score of 6/7 points. In the postpartum period, radical combined radiation therapy was planned but canceled due to bilateral deep venous thrombosis, a floating thrombus in the inferior vena cava and blood clots in the iliac vessels. The patient received a cava filter and underwent anticoagulant therapy. On the 24th day of the postpartum period, a radical hysterectomy with lymphadenectomy was performed due to uterine bleeding.

Patient No. 28 refused early radical treatment and received chemotherapy for Stage IIB cervical cancer with no effect. Due to the tumor growth, a CS with subtotal hysterectomy was performed at GW 36, followed by radiation therapy. A live boy was born weighing 3000g, 50 cm long, with an Apgar score of 7/8 points.

Thyroid lesions (n = 5) were detected in the first stage of the disease. Three out of 5 patients (patients No. 29, 30, 31) were diagnosed with papillary carcinoma at GW 20/21, 22/23 and 27/28, respectively. The diagnosis was confirmed during an ultrasound with the fine-needle aspiration biopsy. Treatment was postponed for the postpartum period. The pregnancies proceeded without complications, and patients were delivered vaginally at term. All children were full-term and healthy. In patient No. 32, the tumor was detected during an ultrasound at GW 8/9. Subsequently, an increase in tumor volume necessitated thyroid gland removal at GW 13/14. Further, the pregnancy proceeded without complications. At 39 weeks, a planned CS was performed (due to 2 previous CS), and a live full-term baby was born. In patient No. 33, thyroid carcinoma was detected at GW 6. Neck MRI performed at GW 17/18 revealed a tumor of the right lobe of the thyroid with a spread to the isthmus and capsule. At GW 22, the thyroid gland was removed together with resection of the muscle layer of the esophagus, chondrolaryngoplasty and microsurgical neurolysis of the recurrent laryngeal nerve. Further, the pregnancy proceeded without complications, and a live full-term baby with an Apgar score of 8/9 points was delivered vaginally at GW 38.

Two pregnant women were diagnosed with kidney neoplasms. In both cases, the disease was detected in Stage I during a planned ultrasound during pregnancy. The first patient was diagnosed with a tumor at GW 18. Treatment was delayed until the postpartum period in the absence of tumor growth. The patient delivered vaginally at GW 39 a live full-term baby (Apgar score – 6/8 points due to fetal hypoxia and vacuum extraction). In the second patient, the neoplasm was detected in the first trimester of pregnancy. Clear cell kidney cancer (T1bN0M0) was diagnosed following the robotic resection of the left kidney at GW 16/17, with no adjuvant therapy needed. The patient delivered vaginally at GW 37, a live full-term baby girl with an Apgar score of 8/9 points.

Two pregnant patients had gastrointestinal neoplasms (colon cancer in 1 case and gastric cancer in another). The first patient underwent surgery for intestinal obstruction at GW 17/18, and sigmoid adenocarcinoma (T3NxM0) was diagnosed. Hartmann abdominal resection of the sigmoid colon and colostomy was done. The delivery was term and an Apgar score of the newborn – 8/9 points. 5 months after delivery, the colostomy was closed during laparoscopy. In the second case, despite the patient’s complaints (even before pregnancy) of epigastric pain, nausea and vomiting, no further examination was prescribed, and only symptomatic treatment was carried out. The diagnosis of gastric cancer was established at GW 29/30 during gastroscopy due to coffee ground vomitus. Subsequent MRI/CT confirmed fundal lesion and enlarged lymph nodes along the lesser curvature. However, no distant metastases were detected. As a result, the diagnosis was gastric cancer cT3NxM0 G3, HER-2/neu negative. The patient was delivered at GW 33 by CS. A live premature baby girl was born weighing 2290g, 43 cm long, and an Apgar score of 7/8 points. The patient immediately began specialized treatment.

In patient No. 38, skin melanoma of the ankle region was diagnosed in the third trimester. At 40 weeks, a planned CS (2 previous CS) was performed with simultaneous skin lesion removal. A live full-term baby was born with an Apgar score of 8/9 points.

Patient No. 39 with retroperitoneal leiomyosarcoma contacted the outpatient ward at GW 27 due to swelling and pain in the right inguinal region. Ultrasound revealed thrombosis of the external iliac vein, common femoral vein, deep femoral vein with signs of initial recanalization. Anticoagulant therapy was beneficial. Two weeks later, liver enzymes were elevated, and the ultrasound of abdomen found lesions within liver parenchyma and inferior vena cava thrombosis. Subsequent MRI demonstrated a retroperitoneal tumor (89 × 70 × 71 mm) with inferior vena cava invasion and moderate portal hypertension, together with multiple liver lesions within both lobes. At GW 32 the patient was delivered via CS due to the increase in the size of the liver, widening diameters of the portal and splenic veins, and enlargement of the retroperitoneal tumor up to 117 × 73 × 73 mm. A live premature baby girl was born with an Apgar score of 7/8 points, a liver biopsy was performed simultaneously, and a morphological diagnosis of leiomyosarcoma was established.

Patient No. 40 with the soft tissue sarcoma of the shoulder had surgery 1.5 months before the pregnancy (excision of the tumor with resection of the shoulder muscle), and adjuvant chemotherapy was recommended, which she refused due to the pregnancy. At GW 22/23, ultrasound/MRI/biopsy confirmed continued tumor growth and 3 courses of chemotherapy were performed according to the VAC-VACA scheme with subsequent delivery at 39 weeks of pregnancy. A live full-term baby was born with an Apgar score of 7/8 points.

CNS tumor was detected in 1 patient. At GW 32, the patient experienced severe headache together with motor aphasia and subsequent generalization. Brain MRI revealed a tumor within the left frontal and parietal lobes that spread to the corpus callosum. The patient was delivered at GW 35/36 via CS. A live premature baby girl was born with an Apgar score of 8/9 points. On the 2^nd^ postpartum day, the patient experienced Jacksonian seizures in the right leg and arm. The patient underwent craniotomy and tumor removal, and the latter turned out to be Grade III anaplastic oligodendroglioma with the *IDH1* (R132H) mutation.

The main data on the management of pregnancy, delivery, and the newborns status in patients with solid neoplasms are presented in Tables 3–4.

### 3.3. Main findings

In our study, the majority of patients (63.4%) received anticancer treatment during pregnancy. Chemotherapy was performed in 19 (46.3%) patients (in 2 cases in combination with surgery), only surgical treatment in 7 (17.1%) patients. In 36.6% of cases, treatment was postponed for the postpartum period.

In most cases, the delivery was at term (n = 28, 68.3%). The majority of the patients (n = 27, 65.8%) delivered vaginally. Fourteen women (34.2%) were delivered via CS, of which – in 4 cases – due to obstetrical complications. In the remaining 10 cases, the indication for surgical delivery was either a worsening of the course of the underlying disease or the need for an earlier delivery to commence antitumor therapy (due to the refusal of women to receive antitumor treatment during pregnancy).

All children born at term (n = 28, 68.3%) were full-term and had no growth restriction, regardless of whether their mothers received treatment during pregnancy or not.

Of the 11 patients with haematological malignancies who received chemotherapy treatment during pregnancy, 5 children were born with an Apgar score of 8/9 points, 1 with 7/8 points. In 4 newborns, lower scores were associated either with or without a complicated obstetric situation during childbirth. In 1 patient, the pregnancy ended with antenatal fetal demise.

In patients with the breast and reproductive organ malignancies, 7 pregnant women received chemotherapy. Of these, 4 pregnant women had children with an Apgar score of 8/9 points, and 3 had 7/7 and 7/8 points, probably due to earlier delivery dates.

In patients with neoplasms beyond the reproductive organs, 5 patients received antitumor treatment during pregnancy, of which only 1 pregnant woman received chemotherapy. All of them have children born at term with an Apgar score of 7/8-8/9 points.

Follow-up of patients postpartum showed that 4 patients with hematologic malignancies and 12 patients with solid tumors had no recurrence of the disease, and their children were in a good state of health. 1 patient with a high-grade ovarian tumor (Patient No. 22) had a tumor recurrence, which was due to the patient’s noncompliance with treatment; the child was healthy. Long-term results showed that 4 out of 16 patients with hematologic cancer, 1 patient with gastric cancer (Patient No. 37) and 1 patient with retroperitoneal sarcoma (Patient No. 39) died. Unfortunately, 18 patients didn’t provide any data.

Our colleagues from the Kulakov Centre for Obstetrics, Gynecology and Perinatology (Moscow, Russia) presented a study including 217 pregnant women with lymphomas and breast cancer diagnosed during pregnancy.^[13]^ Patients were divided into 2 subgroups depending on whether or not they had received chemotherapy treatment during pregnancy. The main finding showed no adverse effect on pregnancy, delivery, or the postpartum period by the use of chemotherapy. A comparative analysis of long-term outcomes of 115 women showed that the 3-year overall survival and relapse-free survival did not differ from those of patients, who were diagnosed out of pregnancy.^[13]^

## 4. Conclusions

In our study, single-site experience in managing pregnant women with different types of cancer and the possible impact of antitumor treatment on the fetus was analysed. Patients with malignancies during pregnancy require a multidisciplinary approach and the use of versatile diagnostic and treatment options; however, possible fetal risks should be taken into consideration.

- Several approaches are to be taken to diagnose a malignancy during pregnancy, which usually includes ultrasound and MRI since they are harmless for baby and mother. In exceptional cases, chest X-rays and CT could be performed with additional measures to protect the uterus. Tumor biopsy provides histological verification and, if it is impossible, such as in the case of ovarian tumor, surgical intervention is performed immediately.
- If a malignant process is suspected and surgical intervention is necessary, it is advisable, if possible, to postpone it beyond GW 15/16.
- The optimal period for laparoscopic access is 14 to 16 weeks of pregnancy. However, a surgical mode is of minor significance.
- If it is necessary to carry out systemic drug treatment, it is possible to start it from the second trimester of pregnancy. The last administration of chemotherapy should be performed 3 weeks before delivery to minimize maternal and fetal complications.

Possible limitations include experience from only 1 medical center, and the fact that not all cancer types were presented, although it follows the natural distribution in the general population.

The management of pregnancy and delivery in this cohort should be carried out in a specialized multidisciplinary medical center with all necessary specialists. Such an approach guarantees conducting a comprehensive clinical examination and prescribing adequate and timely treatment while minimizing the fetal risks. Together this contributes to pregnancy prolongation and creates conditions for the birth of a healthy child.

**Table 3:** Pregnant with malignant tumors of the breast and reproductive organs (n = 8).

No	Diagnosis, stage	Age	Time of diagnosis (weeks of gestation)	Treatment during pregnancy	Labor (weeks)	Mode of delivery	Newborn (Apgar)
17	Breast Cancer II	39	33/34	Not performed	37/38	Spontaneous vaginal	5/7*
18	Breast Cancer III	32	25/26	Refusal of treatment	34/35	Cesarean section	7/8
19	Breast Cancer III	37	20/21	АС N = 2	37/38	Spontaneous vaginal	7/8
20	Breast Cancer III	45	20/21	ЕС N = 4	39/40	Spontaneous vaginal	8/9
21	Breast Cancer III	34	13/14	АС N = 4	38/39	Spontaneous vaginal	8/9
22	Ovarian cancer IIВ	34	24/25	Adnexectomy at GW 25.TCb N = 2	34/35	Cesarean section with hysterectomy and bilateral salpingo-oophorectomy, omentectomy	7/7
23	Ovarian cancer IIВ	29	10/11	Adnexectomy at GW 22.TCb N = 4	38/39	Cesarean section with hysterectomy and bilateral salpingo-oophorectomy, omentectomy	8/9
24	Ovarian cancer IA	34	14/15	Laparoscopy with the left adnexectomy, resection of the omentum, biopsy of the contralateral ovary at GW 22	36/37	Cesarean section (placental abruption)	7/8
25	Ovarian stromal cell tumor IIB	37	12/13	Refusal of treatment	38/39	Spontaneous vaginal delivery. Hysterectomy and bilateral salpingo-oophorectomy on the 8th postpartum day (the tumor continued growth)	8/9
26	Cervical cancer IIВ	34	25/26	TP N = 2	37/38	Cesarean section + radical hysterectomy	8/9
27	Cervical cancer IВ2	41	18/19	Not performed	29/30	Cesarean section with temporary bilateral embolization of the uterine arteries†, radical hysterectomy on the 24th postpartum day	6/7
28	Cervical cancer IIB	33	15/16	ТCb, N = 3	36/37	Cesarean section with temporary bilateral embolization of the uterine arteries, subtotal hysterectomy (the tumortumor continued growth). Radiation therapy in the postpartum period	7/8

AC = doxorubicin+cyclophosphane, EC = epirubicin+cyclophosphane, TCb = paclitaxel+carboplatin, TP = paclitaxel+сisplatin.

* Due to a complicated obstetric situation (the onset of fetal hypoxia, use of the vacuum extractor).

† Preterm delivery due to fetal intrauterine growth restriction, 3rd stage violations of the uteroplacental blood flow and the onset of fetal hypoxia.

**Table 4:** Pregnant with malignant tumors of nonreproductive organs (n = 13).

No	Diagnosis, stage	Age	Time of diagnosis (weeks of gestation)	Treatment during pregnancy	Labour (weeks)	Mode of delivery	Newborn (Apgar)
29	Thyroid cancer I	34	20/21	Not performed	39/40	Spontaneous vaginal	7/8
30	Thyroid cancer I	39	22/23	Not performed	39\40	Spontaneous vaginal	8/9
31	Thyroid cancer I	40	27/28	Not performed	40/41	Spontaneous vaginal	8/9
32	Thyroid cancer I	26	8/9	Thyroidectomy at GW 13/14	39/40	Cesarean section	8/9
33	Thyroid cancer I	27	6/7	Thyroidectomy at GW 22	38/39	Spontaneous vaginal	8/9
34	Kidney cancer I	44	18/19	Not performed	39/40	Spontaneous vaginal	6/8*
35	Kidney cancer I	36	7/8	Robotic resection of the left kidney at GW 16/17	37/38	Spontaneous vaginal	8/9
36	Sigmoid colon cancer рТ3NхM0	37	17/18	Emergency Hartmann abdominal resection of the sigmoid colon at GW 17/18	39/40	Spontaneous vaginal delivery. Closure of the colostomy in 5 months after delivery	8/9
37	Gastric cancer сТ3NxM0	22	29/30	Not performed	33/34	Cesarean section	7/8
38	Skin melanoma of the ankle region T2aNxMx	40	40/41	Not performed	40/41	Cesarean section + skin lesion removal	8/9
39	Retroperitoneal sarcoma IV	31	29/30	Not performed	34/35	Cesarean section	7/8
40	Soft tissue sarcoma of the shoulder	28	22/23	VAC-VACA at 26, 30, and 32 GW	39/40	Spontaneous vaginal	7/8
41	Central nervous system tumor (glioma)	36	32/33	Not performed	35/36	Cesarean section, on the 2nd postpartum day emergency craniotomy and tumor removal	8/9

VAC-VACA = vincristine+doxorubicin+cyclophosphamide+dactinomycin.

* Due to a complicated obstetric situation (the onset of fetal hypoxia, use of the vacuum extractor).
